# Mechanistic study of glutamine metabolic reprogramming driving non-small cell lung cancer progression via the FGF17-FGFR4 axis mediating epithelial-mesenchymal transition

**DOI:** 10.3389/fmolb.2025.1728698

**Published:** 2026-01-02

**Authors:** Qinghua Kong, Xiaoyan Wang, Wei Ding

**Affiliations:** 1 Department of Respiratory and Critical Care Medicine, Dahua Hospital, Xuhui District, Shanghai, China; 2 Department of Pathology, Affiliated Changshu Hospital of Nantong University (Changshu No.2 People’s Hospital), Changshu, China; 3 Department of Respiratory Medicine, Gongli Hospital, Pudong New Area, Shanghai, China

**Keywords:** non-small cell lung cancer (NSCLC), GLUL, FGF17, MEK5/ERK5, epithelial–mesenchymal transition (EMT)

## Abstract

**Introduction:**

The reprogramming of glutamine metabolism holds a pivotal position in the energy provision and biosynthesis of tumors. However, the regulatory mechanism of this phenomenon in non-small cell lung cancer (NSCLC) is still not well-understood. NSCLC is a type of malignancy that has a high incidence and mortality rate globally. There is an urgent need to elucidate the role of glutamine metabolism in its pathological mechanism. This clarification may provide theoretical guidance for developing new therapeutic approaches.

**Methods:**

Core targets of glutamine metabolism were screened by integrating single-cell transcriptomic and RNA sequencing data from public databases. Target expression was validated in clinical samples by immunohistochemistry (IHC) and Western blot (WB), and its association with clinical features was analyzed. Lentiviral gene silencing was employed to establish glutamine-deprived cell models and xenograft mouse models. To evaluate the effects of the target on cell proliferation, redox balance, and migratory/invasive behavior in cell culture and animal models, we utilized Transwell assays, colony formation assays, redox detection kits, and Seahorse metabolic flux analysis. Subsequently, WB and IHC served to elucidate the downstream pathways and potential synergistic effects of the drugs.

**Results:**

Analysis of the single-cell atlas revealed a marked increase in epithelial (Epi) cell populations in the tumor milieu of NSCLC. By integrating weighted gene co-expression network analysis (WGCNA) with RNA sequencing, fibroblast growth factor 17 (FGF17) was pinpointed as a crucial regulatory factor. High FGF17 expression showed a strong association with poor prognosis in patient (*p* = 0.0078). Consistent clinical data further demonstrated that FGF17 upregulation was associated with higher TNM stages and the presence of lymph node metastasis. Functional and mechanistic analyses revealed that silencing FGF17 suppressed the FGFR4/MEK5/ERK5 signaling cascade, disturbed NRF2-dependent redox homeostasis, and consequently impaired epithelial–mesenchymal transition (EMT), leading to a marked reduction in cancer cell motility and invasiveness. *In vivo*, targeting FGF17 was shown to synergistically enhance cisplatin antitumor activity and reverse the EMT phenotype.

**Conclusion:**

As a critical driver of glutamine metabolic reprogramming, FGF17—activated under conditions of GLUL overexpression—stimulates the FGFR4/MEK5/ERK5/NRF2 signaling cascade to maintain redox homeostasis and promote invasion, thereby accelerating NSCLC progression. Targeted intervention of the pathway reverses malignant phenotypes and enhances chemosensitivity. These findings highlight FGF17 as a potential therapeutic target for NSCLC and provide new insights into tumor metabolism and EMT, thereby may paving the way for novel combination therapies.

## Introduction

Non-small cell lung cancer (NSCLC), accounting for approximately 80%–85% of all lung cancer cases, represents the dominant histopathological form of the disease and remains the leading contributor to cancer mortality worldwide (Siegel et al., 2025). According to their pathological features, NSCLC is categorized into lung adenocarcinoma (LUAD), lung squamous cell carcinoma (LUSC), and large cell lung carcinoma (LCLC) (Duma et al., 2019). The etiology of NSCLC involves multifactorial interactions, including tobacco exposure, environmental pollutants (e.g., PM2.5, radon), occupational carcinogen exposure, and genetic susceptibility (Chen et al., 2022). Epidemiological evidence shows a continuous global increase in lung cancer cases and deaths, with the most pronounced rise observed in developing nations such as China, where this malignancy poses a significant challenge to public health (Chen et al., 2022). Presently, in clinical settings, various multimodal treatment approaches are being utilized. These include surgical procedures, radiation therapy, chemotherapy, precision-targeted therapies, and immune checkpoint-blocking agents. Despite advances in treatment, the 5-year overall survival for individuals diagnosed with non-small cell lung cancer (NSCLC) remains under 23%. Specifically, for those patients diagnosed at advanced stages, the survival rate drops to less than 5% (Osmani et al., 2018; Sorin et al., 2024; Rahal et al., 2025). Therapeutic bottlenecks arise, on one hand, from the immunosuppressive properties of the tumor microenvironment that compromise drug efficacy, and on the other, from drug toxicity and the development of resistance (Reck et al., 2022). Therefore, additional studies are required to facilitate the development of novel therapeutic strategies.

Metabolic reprogramming is a hallmark of tumor cells. Glutamine, the most abundant non-essential amino acid in plasma, plays a crucial role in tumor metabolism (Yoo et al., 2020; Ma et al., 2022). In the tricarboxylic acid (TCA) cycle, glutamine undergoes deamination by glutaminase (GLS) to generate glutamate, thereby providing a carbon source for tumor cells. Additionally, through anaplerosis, glutamine replenishes intermediates of the TCA cycle. Moreover, glutamine serves as a nitrogen source for nucleotide synthesis. Studies have demonstrated that glutamine contributes to glutathione synthesis to maintain redox homeostasis, a function closely associated with the development of chemoresistance (Rouse et al., 1995). In many cancer types, tumor cells exhibit increased dependence on glutamine, a phenomenon referred to as “glutamine addiction” (Yoo et al., 2020). Analysis of over 80 NSCLC cell lines revealed that proliferation rate positively correlated with glutamine consumption (Chen et al., 2019). Furthermore, studies have shown that glutamine deprivation attenuates cisplatin resistance in chondrosarcoma by suppressing amphiregulin expression, which consequently inhibits NADPH generation and promotes ROS accumulation (Wu et al., 2023). This adaptive metabolic response satisfies the energy demands of tumor proliferation and invasion, conferring a survival advantage under hypoxic, nutrient-deprived, and drug-stressed microenvironments (Chakraborty et al., 2021; Ma et al., 2022). In addition, glutamine metabolism interacts with various intracellular signaling pathways to maintain malignant phenotypes. Although it is well established that NSCLC exhibits a marked dependency on glutamine metabolism, the mechanistic link between aberrant activation of glutamine metabolism and malignant transformation remains incompletely understood (Zhou et al., 2023). Moreover, the intricate crosstalk between glutamine metabolic rewiring and various oncogenic signaling pathways in NSCLC cells has yet to be fully elucidated. Compellingly, enhanced glutamine metabolism represents a key contributor to therapeutic failure and drug resistance across multiple treatment modalities. Recent studies have revealed that inhibiting the glutamine transporter SLC1A5 can sensitize NSCLC cells to epidermal growth factor receptor-tyrosine kinase inhibitors (EGFR-TKIs) and delay the acquisition of resistance (He et al., 2021; Pan et al., 2022). Therefore, a deeper understanding of the functional roles and regulatory mechanisms underlying glutamine metabolic reprogramming in NSCLC may provide a critical scientific foundation for the development of novel targeted therapeutic strategies.

The gene encoding Glutamate-Ammonia Ligase (GLUL) produces glutamine synthetase (GS), the only enzyme responsible for catalyzing glutamine synthesis *in vivo*. In NSCLC, GS is upregulated to maintain intracellular glutamine levels. Suppressing GS expression decreases the growth of lung cancer cell lines, patient-derived organoids, and xenograft models, and simultaneously enhances the effectiveness of microtubule-targeting drugs (Tang et al., 2021; Zhao et al., 2022). It has also been demonstrated that GS deficiency in macrophages promotes vascular normalization and suppresses tumor cell migration (Palmieri et al., 2017). In certain cancer treatments, GS expression has been linked to therapeutic resistance. In gefitinib-resistant cells, GS levels are reduced, whereas GS expression is elevated in sensitive cells, suggesting the potential of GS as a predictive biomarker of therapeutic response (Wang et al., 2018). Nevertheless, the interaction network between GS, the tumor metabolic microenvironment, and signaling pathways remains unresolved, and the precise molecular mechanism by which glutamine metabolism regulates NSCLC proliferation, migration, and resistance through GS has yet to be elucidated.

Based on these observations, a multidimensional research strategy was implemented in this study: bioinformatic analysis of public databases identified potential key targets of glutamine metabolism in NSCLC, which were subsequently validated clinically and functionally using both cell-based and animal experiments. Integration of bioinformatics and literature review was further used to elucidate the association between glutamine metabolism and NSCLC progression. The study aims to uncover the dynamic regulatory mechanism of GLUL in glutamine metabolism within NSCLC, with the potential to overcome current therapeutic bottlenecks, provide novel evidence for targeted strategies against glutamine metabolism, and contribute to a deeper mechanistic understanding of lung cancer.

## Materials and methods

### Data acquisition and processing

Data from single-cell sequencing (scRNA–seq, GSE19804 dataset) and microarray (GSE131907 dataset) related to LUAD and Lung Carcinoma sourced from the Gene Expression Omnibus (https://www.ncbi.nlm.nih.gov/geo). The GSE19804 dataset comprises samples exclusively from 120 non-smoking female lung adenocarcinoma patients in Taiwan, China, while the GSE131907 dataset includes 58 lung adenocarcinoma samples sourced from the College of Medicine, the Catholic University of Korea. Moreover, information regarding tumor characteristics and patient prognoses for NSCLC specimens was also collected. From the GSE19804 dataset, 60 disease group samples (NSCLC-Tumor) and 60 normal group samples (NSCLC-Normal) were selected. The GSE131907 dataset included 11 disease group samples (Lung-T) and 11 normal group samples (Lung-N). The levels of expression of the genes of interest in both normal and cancerous tissues were measured. An analysis using the Kaplan-Meier curve for the targets identified earlier was carried out through the Kaplan-Meier Plotter website (https://kmplot.com/). As the GEO database provides publicly available data, this study did not require ethical approval or informed consent.

### Patients and clinical samples characteristics

The clinicopathological information was obtained from the medical archives at the Pathology Department of Changshu No.2 People’s Hospital, located in Changshu, Jiangsu Province. The patients were chosen according to the condition of not having undergone any preoperative chemotherapy or radiotherapy. Before the collection of samples, written informed consent was acquired from every patient. Based on the staging standards of the 2024 Chinese Society of Clinical Oncology (CSCO) Guidelines for NSCLC, this research recruited 87 patients with pathologically verified NSCLC. Among these patients, 8 were stage I, 29 stage II, 12 stage III, and 38 stage IV. For analytical purposes, stages I–II were categorized as early-stage NSCLC (early NSCLC), while stages III–IV were classified as advanced-stage disease (advanced NSCLC). Additionally, adjacent non-tumorous tissues from 39 randomly selected patients served as the control group (Control). All diagnoses were histopathologically validated. The study protocol received approval from the Institutional Review Board of Changshu No.2 People’s Hospital prior to implementation (2019LYPNo.025).

### Immunofluorescence staining

Tumor tissues underwent a series of processing steps for subsequent analysis. First, they were immersed in phosphate-buffered saline (PBS) and rinsed three times, with each rinse lasting 2 min. After that, the tissues were fixed in ice-cold methanol at a temperature of-20 °C for 30 min. Following fixation, the methanol was carefully discarded, and PBS was used to wash the cells. This second round of washing consisted of three cycles, each lasting 5 min. Following the PBS washes, the samples were treated with a 0.1% Triton X-100 solution. They were incubated at room temperature for 15 min to permeabilize the cell membranes. After the incubation, another set of three PBS washes, each 5 min long, was carried out to remove the Triton X-100. The samples were blocked with bovine serum albumin (BSA) for 30 min to reduce non-specific interactions. Subsequently, primary antibodies targeting FGF17 were applied. The primary antibodies were prepared at a 1:100 dilution PA5-109722 from Thermo. To allow antigen–antibody binding, the samples underwent incubation at 37 °C for 60 min. After exposure to the primary antibodies, PBS washing was performed three times, each lasting 5 min. Subsequently, a goat-derived anti-rabbit IgG antibody was introduced as the secondary reagent. The secondary antibody, with a product number of A-11008 from Thermo, was used at a dilution of 1:500. Incubation was performed at 37 °C for 60 min in the dark to allow the secondary antibody to bind to the primary antibody. Following secondary antibody incubation, the samples were washed three times with PBS, each wash lasting 5 min. Then, the samples were stained with a DAPI solution (product number C1314L from Beyotime) for 10 min to label the cell nuclei. After staining, three additional PBS washes were performed to eliminate any excess dye. Finally, the samples were prepared for imaging. Twenty microliters of mounting medium (product number P0131-25 mL from Beyotime) were applied to the slides, which were then sealed. When the mounting medium was completely dry, fluorescence images were obtained under a fluorescence microscope.

### Construction of lentiviral vectors

The lentiviral knockdown vector, pLKO.1-puro (shRNA vector, TR30021, Thermo), carrying the shRNA sequence targeting fibroblast growth factor 17 (shFGF17), was purchased. In NSCLCH1299 cells (NCI-H1299, CL-0165, Procell), the Lentivirus Packaging Mix (A35684CN, Invitrogen) was employed to encapsulate this vector into lentiviral particles. Forty-eight hours subsequent to transfection, the supernatant that held the lentiviruses was gathered. The resulting titer of the collected supernatant was 1.2 × 10^8^ TU/mL.

### Cell culture

The NCI-H1299 cell line was obtained from Wuhan Procell Biotechnology Co., Ltd. (CL-0165). The cells were cultivated in RPMI 1640 culture medium (product numbers #15-040-CV and #10-040-CV, Corning). This medium was supplemented with 10% fetal calf serum (A5669701, Gibco) and 1% penicillin/streptomycin solution (product number #15070063, Gibco). Subsequently, the cells were cultured at 37 °C in a 5% CO_2_ atmosphere. Stable shFGF17 cells were generated using lentiviral transduction of pLKO.1-puro constructs containing validated shRNA sequences and maintained under 2 μg/mL puromycin selection.

For the experimental treatments, cells were distributed at 5 × 10^4^ cells per square centimeter across 6-well culture plates. After 24-h attachment, five treatment groups were established: Control (Ctrl): untreated. Glutamine deprivation (-Gln): cells in glutamine-deficient RPMI-1640 (15-040-CV, Corning). FGF17 knockdown (shFGF17): shFGF17 cells in complete RPMI-1640 (10-040-CV, Corning). Combinatorial treatment (-Gln + shFGF17): shFGF17 cells in glutamine-deficient medium. Metabolic rescue (-Gln + shFGF17 + α-KG): shFGF17 cells in glutamine-deficient medium supplemented with 2 mM α-ketoglutarate (#75890, Sigma).

### Cell viability measurement

Statistical analysis of cell viabilities was carried out using the Cell Counting Kit-8 (CCK-8; CA1210, Solarbio). During the logarithmic growth phase, NCI-H1299 cells were distributed into 96-well plates at a density of 8,000 cells per well. These cells were then cultivated in RPMI-1640 medium (#15-040-CV, Corning) supplemented with 10% fetal bovine serum (FBS) and different concentrations of L-glutamine (G8540, Sigma). The L-glutamine concentrations used were 0, 0.5, 1, 2, and 4 mM. Culture conditions were set at 37 °C with 5% CO_2_. After 24 and 48 h of treatment, 10 μL of the CCK-8 reagent was introduced into each well. A 2-h incubation at 37 °C was performed on the plates. Measurement of absorbance at 450 nm was carried out with a microplate reader (BioTek Synergy H1).

### EdU assay

The initial step was to seed the NCI-H1299 cells in 24-well plates (5 × 10^4^ cells/well) and perform different operations according to the experimental groups. After 24 h, EdU labeling was performed using 10 μM Click-iT™ EdU reagent (C10639, Thermo) for 2.5 h. A 15-min fixation of the cells was performed using 4% paraformaldehyde (PFA). After fixation, the cells underwent two rounds of washing with phosphate-buffered saline (PBS). Following this, they were made permeable by exposure to 0.3% Triton™ X-100 for 10 min. Following permeabilization, additional PBS washes were performed on the samples. Blocking was performed with 3% bovine serum albumin (BSA) in PBS for 30 min to reduce non-specific binding. After blocking, the samples were incubated with the Click-iT™ reaction mixture for 30 min in the dark. Next, the nuclei were stained as a counterstain using 1 μg/mL 4′,6-diamidino-2-phenylindole (DAPI) for 10 min. After the staining procedure, imaging and photography of the cells were performed with a Nikon Eclipse Ti2 microscope using NIS-Elements v5.30 software. To determine the quantity of EdU^+^/DAPI^+^ cells, ImageJ software was employed. All experiments were conducted in triplicate to confirm the robustness and reproducibility of the findings.

### Transwell

The matrigel gel (product number #354480, Corning) was combined at a 1:1 ratio with ice-cold, serum-free RPMI-1640 medium at 4 °C. Fifty microliters of this resulting mixture were then introduced into the upper compartment of each Transwell plate. Subsequently, the plate was placed at 37 °C for 2 h to allow the mixture to polymerize. Next, the upper chambers received cells (a quantity of 5 × 10^4^ suspended in 200 μL of serum-free medium). Concurrently, the lower chamber was filled with 800 μL of medium supplemented with 20% fetal bovine serum (FBS). After incubating the setup for 24 h, the non-invading cells were eliminated by washing the chambers with phosphate-buffered saline (PBS). The cells that had invaded were then fixed using 4% paraformaldehyde for a duration of 10 min. Following fixation, they were stained with 0.1% crystal violet (product code C0775, Sigma) for 30 min. After two rinses with PBS, the cells were examined, photographed, and quantified with an inverted microscope. The cell counting process was carried out in at least four randomly chosen microscopic areas. After extraction of crystal violet with 10% acetic acid (200 μL per well), absorbance readings at 570 nm were obtained from the solution. Three independent repetitions of the entire experimental procedure were performed.

### Colony formation analysis

NCI-H1299 cells were plated into 6-well plates at a concentration of 800 cells per well. After a 14-day cultivation period, the cells were fixed with 4% paraformaldehyde. Subsequently, they were rinsed with phosphate-buffered saline (PBS). After that, the cells were dyed with 0.5% crystal violet (C0775, Sigma). Finally, images of the stained cells were captured using a camera.

### Animal model establishment

Thirty-two female NOD/SCID mice, aged 5 weeks and weighing 20 g plus or minus 2 g, were procured from Beijing Weitonglihua Experimental Animal Technology Co., Ltd. The mice were kept in a Specific Pathogen Free (SPF)-grade facility, with environmental conditions of 60%–65% humidity and 22 °C–25 °C temperature. The study was conducted following experimental guidelines and received approval from the Institutional Animal Ethics Committee of Shanghai 10th People’s Hospital (SHDSYY-2024-3031-3).

After 1 week of adaptation, the experiment began. Fresh tumor tissues from treatment-naïve advanced NSCLC patients were obtained. Necrotic regions and fibrous capsules were dissected on ice, and tissues were minced into 15 mm^3^ fragments in ice-cold PBS. Fragments were loaded into 16-gauge trocars and subcutaneously implanted into the right flank (1-2 sites per mouse). Tumor growth was monitored twice weekly until volumes reached approximately 150 mm^3^ (calculated as V = 0.5 × L × W^2^). Mice were randomized into four treated groups. PDX group: intraperitoneal (i.p.) 0.9% saline every 72 h; Cisplatin monotherapy group: i.p. Injection of cisplatin (5 mg/kg, dissolved in 0.9% saline; #S1166, Selleckchem) every 72 h; shFGF17 group: intratumoral injection of shFGF17 lentivirus (2 × 10^7^ TU, pLKO.1-puro, TRCN0000423211) weekly; Combination therapy group: i.p. Injection of cisplatin (5 mg/kg every 72 h) plus intratumoral injection of shFGF17 lentivirus (2 × 10^7^ TU weekly).

Tumor dimensions were measured using digital calipers every 3 days. On day 28 post-randomization, mice were deeply anesthetized via inhalation of 2% isoflurane for >2 min (fresh gas flow rate: 4 L/min). Subsequently, blood samples were collected immediately, followed by euthanasia via cervical dislocation. Researchers harvested the tumors, weighed them, and stored them at −80 °C.

### Assessment of metabolic and redox parameters

Commercialized diagnostic test kits, including fluorogenic probe H2DCFDA (HY-D0940, MCE), GSH/GSSG Assay Kit (HY-K0311, MCE), JC-1 mitochondrial membrane potential assay kit (HY-K0601, MCE), were employed to assess metabolic and redox parameters in NCI-H1299 cells or mice tutors tissue homogenates. Furthermore, the Seahorse XF Pro Analyzer (Agilent) was employed to gauge the oxygen consumption rate (OCR) and extracellular acidification rate (ECAR) in NCI-H1299 cells. All assays were performed according to the manufacturers’ protocols with at least three technical replicates per condition and repeated in six independent biological experiments.

### Western blot (WB) analysis

RIPA lysis buffer (P0013E, Beyotime) supplemented with PMSF (ST506, Beyotime) was employed to extract the total protein from clinical specimens, NCI-H1299 cells, and mouse tumors. A BCA assay kit (P0009, Beyotime) was utilized to measure protein levels. The samples were then denatured by being boiled in 5×SDS loading buffer (AM8547, Thermo) at 95 °C for 10 min. Separation of approximately 30 μg of total protein per lane was performed using 10% SDS-polyacrylamide gel electrophoresis (SDS-PAGE) (PG112, Epizyme). Transfer of the separated proteins onto polyvinylidene fluoride (PVDF) membranes (IPVH00010, Millipore) was performed. Blocking of the membrane with 5% non-fat milk for 1 h at room temperature was performed to prevent non-specific binding. Incubation of the membrane with primary antibodies was performed overnight at 4 °C. Primary antibody usage and their dilutions are listed below: E-cadherin (1:2000; #3195, CST), Vimentin (1:2000; #5741, CST), GLUL (1:1000; #80636, CST), FGF17 (1:1000; PA5-109722, Thermo), FGFR4 (1:1500; #8562, CST), MEK5 (1:1500; #40473, CST)、phospho-MEK5 (Thr) (1:1000; PA5-105899, Thermo), ERK5 (1:1500; #12950, CST), phospho-ERK5 (Thr219, Tyr221) (1:1000; PA5-114573, Thermo), NRF2 (1:1000; #12721, CST), and GAPDH (1:1000; MA5-15738, Thermo). After three 10-min washes with TBST, horseradish peroxidase (HRP)-gated goat anti-rabbit IgG H&L secondary antibody (1:2000; ab97051, Abcam) was applied to the membranes for 1 h at room temperature. After conducting TBST washes, the protein bands were made visible via a Bio-Rad imaging system. Subsequently, ImageJ software was used to analyze the protein bands. To ensure reliability, every experiment was carried out three times.

### Statistical analysis

A two-way analysis of variance (ANOVA) was carried out, followed by Tukey’s *post hoc* test for multiple comparisons. These statistical procedures were executed using SPSS version 26.0, a product of IBM in the United States. The data was then visualized with GraphPad Prism 8.0.5. Calculation of Pearson’s or Mander’s correlation coefficients was performed for the immunofluorescence datasets. Presentation of the data is expressed as mean values with their corresponding standard deviation (SD). Error bars on the visualizations signify the SD. Statistical significance is denoted as follows: * indicates *p* < 0.05, ** indicates *p* < 0.01, and *** indicates *p* < 0.001. All the experiments incorporated a minimum of three biological replicates, where *n =* 3.

## Results

### scRNA-seq and RNA-seq reveal the key roles of glutamine metabolism reprogramming in NSCLC: FGF17 as a potential therapeutic target

To clarify the cellular diversity and molecular traits of NSCLC, a multi-omics method that combines sscRNA-seq and bulk RNA sequencing was employed. This approach facilitated the discovery of genes with differential expression (DEGs). By merging the data from these two sequencing platforms, the crucial genes associated with glutamine metabolic reprogramming in the tumor microenvironment were identified.

An examination of the public databases GSE19804 and GSE131907 indicated that in the majority of cells, the mitochondrial percentage (percent_mito) was less than 20%, the ribosomal percentage (percent_ribo) was less than 60%, and the hemoglobin percentage (percent.hb) was less than 1% (Supplementary Figure S1A). Afterward, cells of poor quality were eliminated by applying quality control parameters. These criteria included an RNA feature count (nFeature_RNA) of less than 6000, a minimum cell count (min.cells) of 5, a minimum number of features (min.features) of 300, a mitochondrial percentage (percent_mito) of less than 20%, a ribosomal percentage (percent_ribo) greater than 3%, and a hemoglobin percentage (percent_hb) of less than 1%. Consequently, an expression matrix was generated, encompassing 14,735 genes and 21,549 cells. A correlation assessment of the sequencing depth was conducted. The analysis showed that the correlation coefficient (r value) between the RNA count (nCount_RNA) and the RNA feature count (nFeature_RNA) was 0.93 (Supplementary Figure S1B). This high correlation value suggests that the data quality is satisfactory and appropriate for subsequent in-depth analysis.

**FIGURE 1:**
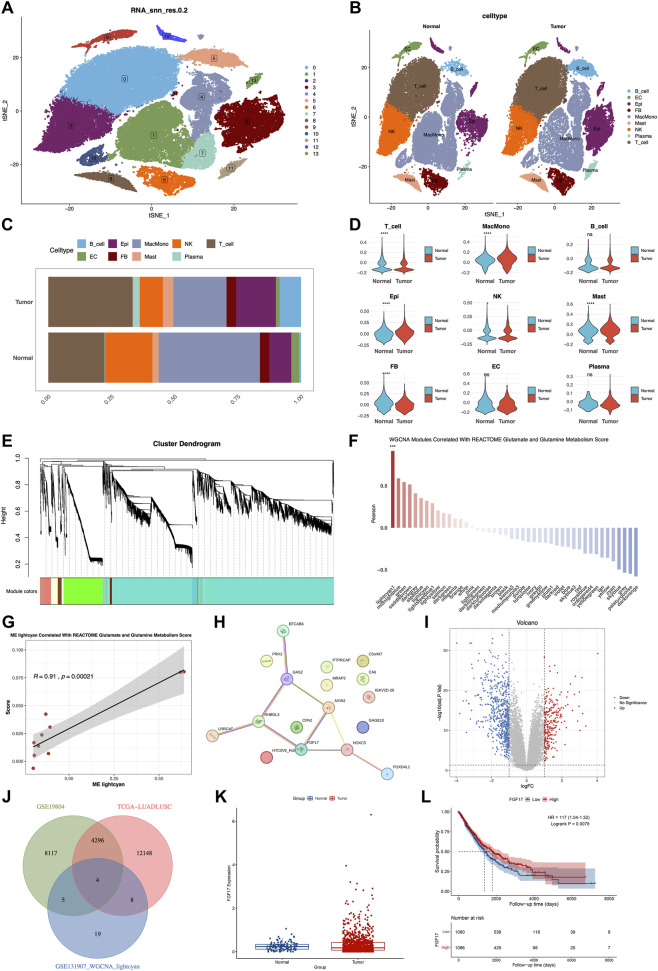
Cell clustering and communication analysis of scRNA-seq data. **(A)** tSNE visualization of cancer cell clusters, the cell types corresponded to cell types shown in **(B)**; **(B)** tSNE plot stratified by tissue origin (normal vs. tumor), highlighting differential cell distribution; **(C)** Proportions of cell subpopulations; **(D)** T-test comparing cell proportions between normal and NSCLC samples; **(E)** Dendrogram of DEGs clustered by dissimilarity (1−TOM); **(F)** WGCNA modules correlated with Reactome glutamate/glutamine metabolism score; **(G)** ME lightcyan module association with glutamate/glutamine metabolism score; **(H)** PPI network of DEGs; **(I)** Volcano plot of RNA-seq DEGs; **(J)** Overlap of DEGs from RNA-seq and scRNA-seq epithelial cells; **(K)** FGF17 protein expression in normal vs. tumor samples; **(L)** Kaplan–Meier survival analysis of FGF17 expression and overall survival in NSCLC.

Subsequently, the UMAP algorithm was utilized to conduct nonlinear dimensionality reduction on the top 20 principal components. The cluster package was then used to visualize clustering patterns at various resolutions (Supplementary Figure S1C). Through an in-depth UMAP clustering analysis, all cells were grouped into 13 clusters (Figure 1A). Using lineage-specific marker genes obtained from relevant literature and the CellMarker database, nine distinct cell types were identified: B lymphocytes (B_cell), endothelial cellular elements (EC), epithelial cellular entities (Epi), fibroblast cells (FB), a combined group of macrophages and monocytes (MacMono), mastocytes (Mast), natural killer lymphocytes (NK), plasmocytes (Plasma), and T lymphocytes (T_cell) (Figure 1B). Moreover, the UMAP expression profiles of the marker genes corresponding to these 9 cell types were presented (Supplementary Figure S1D). A detailed account of the composition and distribution of these 9 cell types within the samples was presented. T-tests were applied to contrast the disparities in cell quantities between normal samples and NSCLC samples. The analysis revealed that, relative to normal samples, MacMono, Epi, NK, and Mast cell counts were significantly increased, while T and FB cell counts were significantly decreased (Figures 1C,D).

The epithelial cell (Epi) subpopulation was extracted for weighted gene co-expression network analysis (WGCNA) to uncover genes co-expressed in relation to glutamine metabolism. Clustering analysis revealed that one outlier sample was identified and removed using a cut-height threshold of 1,000,000 (Supplementary Figure S1E). The optimal soft-thresholding power 10 was established using the scale-free topology fit index (*R* ^2^ = 0.80) and average connectivity criteria (Supplementary Figure S1F–G). Thereafter, 44 gene modules were obtained (Figure 1E). Among these modules, the light-cyan1 module was chosen as the crucial module. It had a Pearson correlation coefficient where *r* = 0.91 and *p* = 0.00021 (as shown in Figures 1F,G). Seventeen genes within this module were identified as hub genes. Protein-protein interactions were retrieved using the STRING database. Subsequently, through hubba analysis in Cytoscape, the top eight hub genes were determined (Figure 1H; Supplementary Table S1).

Comparison of gene expression in epithelial cells from adjacent normal tissues and NSCLC samples revealed 341 differentially expressed genes (DEGs). Among these, 147 genes were notably upregulated, while 194 genes were significantly downregulated (Figure 1I). By intersecting DEGs from GSE19804 (*p* < 0.05) and TCGA LUAD/LUSC (Padj <0.05 and |log2FoldChange| ≥ 1) with single-cell lightcyan1 module genes, four overlapping genes were obtained: FGF17, DLG1-AS1, RHBDL3, and CA6 (Figure 1J). Based on the score values in the hubba analysis table, FGF17 was chosen for subsequent investigation. The analysis of the protein expression patterns of FGF17 in both normal and tumor tissues was carried out by utilizing the human protein atlas (HPA) database. The findings indicated that the expression of FGF17 was notably higher in ovarian cancer tissue when compared to normal lung tissue (Figure 1K). To further investigate the prognostic role of this key gene in NSCLC, patients were divided into high- and low-expression groups. Proportional hazards assumption testing, survival regression model fitting, and Kaplan-Meier curve construction were carried out. The results showed that patients with high FGF17 expression had significantly worse survival than those with low expression (HR = 1.17, 95% CI: 1.04–1.32, *p* = 0.0078, Figure 1L). Thus, FGF17 was identified as a glutamine metabolism-related potential therapeutic target significantly associated with NSCLC.

### Validation of FGF17 involvement in NSCLC using clinical samples

**FIGURE 2:**
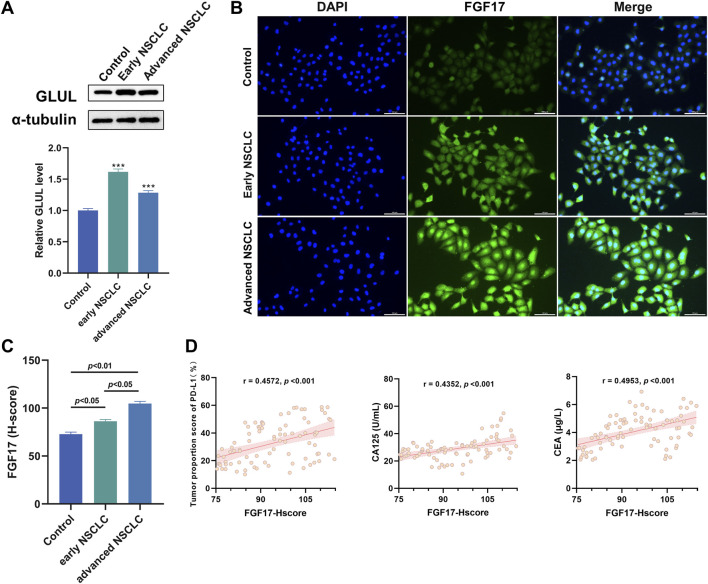
Verification of the role of FGF17 protein in NSCLC in clinical samples. **(A)** The changes in GLUL protein expression in NSCLC patients were evaluated through Western blot analysis; **(B)** Immunofluorescence assay of FGF17 protein in clinical samples (*in vitro* scale bar = 100 μm); **(C)** Quantitative results of FGF17 immunofluorescence by ImageJ (t-Test); **(D)** Correlation analysis of PD-L1, CA125, and CEA with the expression level of RPL11 protein (H-score). (Quantitative data are presented as the Mean plus or minus the Standard Deviation, with a minimum of three samples per group. When compared to the Control group, highly significant differences were observed with *** indicating *p* < 0.001).

To better elucidate the regulatory relationship between GLUL and FGF17 in NSCLC, WB was employed to assess GLUL expression in normal and NSCLC samples. The results showed that GLUL expression was significantly higher in advanced NSCLC samples than in normal counterparts. This means that the degree to which glutamine synthesis is activated in NSCLCis quite obvious (Figure 2A, *p* < 0.001). This finding confirmed that NSCLC tissues depend on glutamine metabolism. Subsequently, immunofluorescence staining was used to assess FGF17 expressions in normal and NSCLC samples. The results indicate that FGF17 expression is significantly elevated in both early and advanced NSCLC compared to normal samples. This increase in expression shows a positive association with NSCLC progression (Figures 2B,C). The relationship between FGF17 expression and clinical characteristics was evaluated using H-scores obtained from immunofluorescence staining. The clinical indicators considered here comprised PD-L1-positive cell fraction in tumor tissues and serum levels of carbohydrate antigen 125. That is CA125, as well as carcinoembryonic antigen (CEA). Correlation analysis demonstrated that FGF17 H-scores were significantly associated with all these indicators (Figure 2D).

### shFGF17 inhibits migration and invasion of NSCLC cells under glutamine deprivation

**FIGURE 3:**
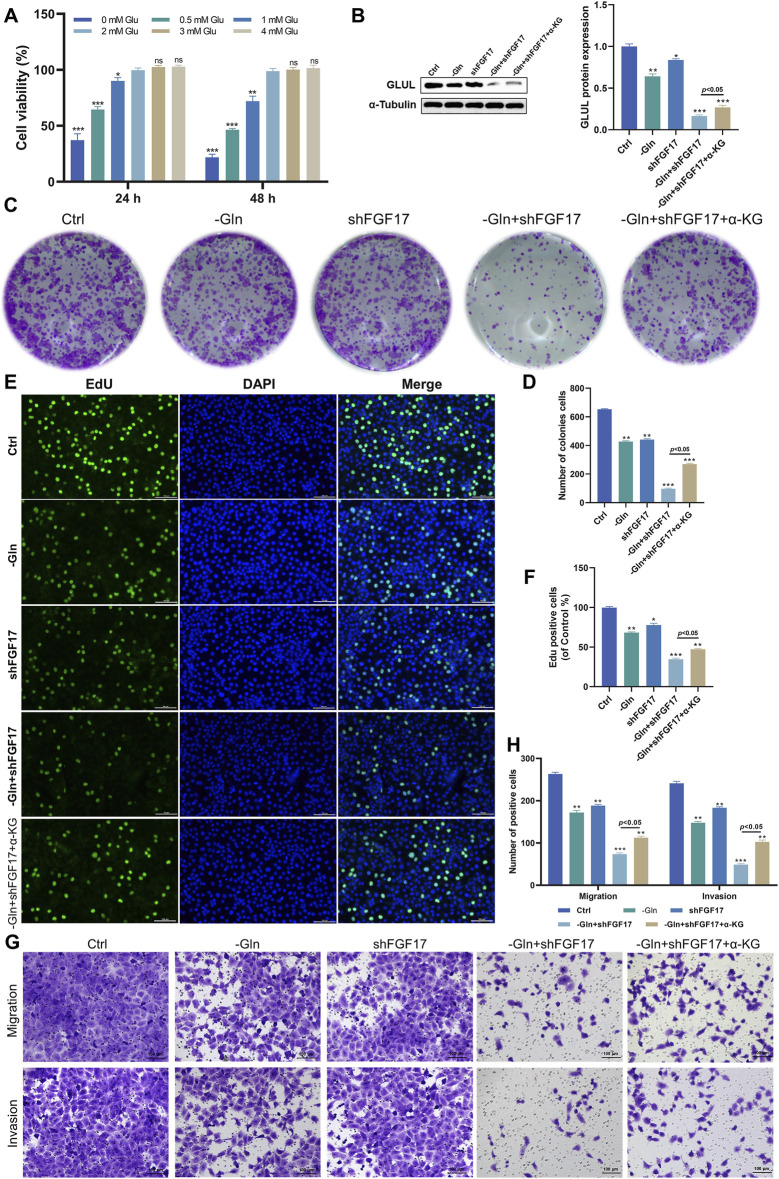
Glutamine metabolism achieves the effects on the proliferation and migration of NCI-H1299 cells through FGF17. **(A)** The CCK-8 test was employed to gauge the impacts of various glutamine concentrations and incubation durations on the viability of NCI-H1299 cells. **(B)** Western blot examination was carried out to evaluate the protein expression of GLUL. **(C)** A colony formation test was conducted on NCI-H1299 cells. **(D)** The colony formation experiment’s quantitative outcomes were analyzed using ImageJ. **(E)** An EdU test was performed to assess the impact of FGF17 silencing and glutamine deficiency on the proliferative capacity of NCI-H1299 cells (scale bar = 100 μm). **(F)** The quantitative results of the EDU test were obtained via ImageJ. **(G)** A Transwell test was utilized to ascertain the effects of FGF17 silencing and glutamine deprivation on the migratory and invasive capabilities of NCI-H1299 cells (scale bar = 100 μm). **(H)** The quantitative results of the Transwell test were determined using ImageJ. (Quantitative data are expressed as Mean ± SD, with three samples at least per group. For comparisons with Ctrl group, *p < 0.05, **p < 0.01, ***p < 0.001).

This study aims to explore how FGF17 expression affects migration of NSCLC tumor cells. Firstly, a cell counting Kit-8 assay was conducted. Specifically, NCI-H1299 cells were cultured in an environment with gradient glutamine concentrations, and then the proliferation activity of these cells was quantitatively evaluated. Identify the critical glutamine deprivation concentration that can significantly inhibit tumor growth to establish a standardized metabolic stress cell model. As shown in Figure 3A, when glutamine concentration exceeded 2 mM, no significant changes in NCI-H1299 cell viability were observed. At 1.0 mM glutamine, the proliferation rate was 90.14% ± 1.93% at 24 h (p < 0.05) and decreased to 71.92% ± 4.54% at 48 h (p < 0.05). Cell proliferation was thus significantly suppressed at 48 h. Therefore, 1 mM glutamine for 24 h was chosen as the glutamine deprivation condition. WB was employed to assess GLUL protein levels in the Ctrl and -Gln groups, aiming to examine the effect of glutamine deprivation on tumor cell metabolism. The obtained results confirmed that when 1 mM of glutamine was deprived, it could effectively disrupt the metabolism of NSCLC cells (Figure 3B). Thereafter, FGF17-overexpressing NCI-H1299 cells were generated using lentiviral transfection for subsequent experiments. In this paper, the colony formation (Figures 3C,D) and EdU methods (Figures 3E,F) were used to determine the cell aggregation ability and cell proliferation ability. From the obtained results, after comparison with the control group, it was found that the colony formation and cell proliferation of NCI-H1299 cells in the -Gln + shFGF17 group were significantly reduced. However, the aggregation ability and cell proliferation ability of NCI-H1299 cells in the α -KG group showed a very prominent recovery (Figures 3C–F). Transwell assays further revealed that–Gln and shFGF17 each significantly inhibited migration and invasion of NCI-H1299 cells, while α-KG supplementation partially reversed these effects in the–Gln + shFGF17 group (Figures 3G,H). This indicates that FGF17 facilitates the migration and invasion of cancer cells through sustaining glutamine metabolism and supporting α-KG-driven TCA cycle activity, further confirming its intervention in metabolic reprogramming during NSCLC progression.

### Glutamine metabolism promotes NSCLC progression by modulating oxidative stress and epithelial–mesenchymal transition via the GLUL–FGF17–FGFR4 axis

**FIGURE 4:**
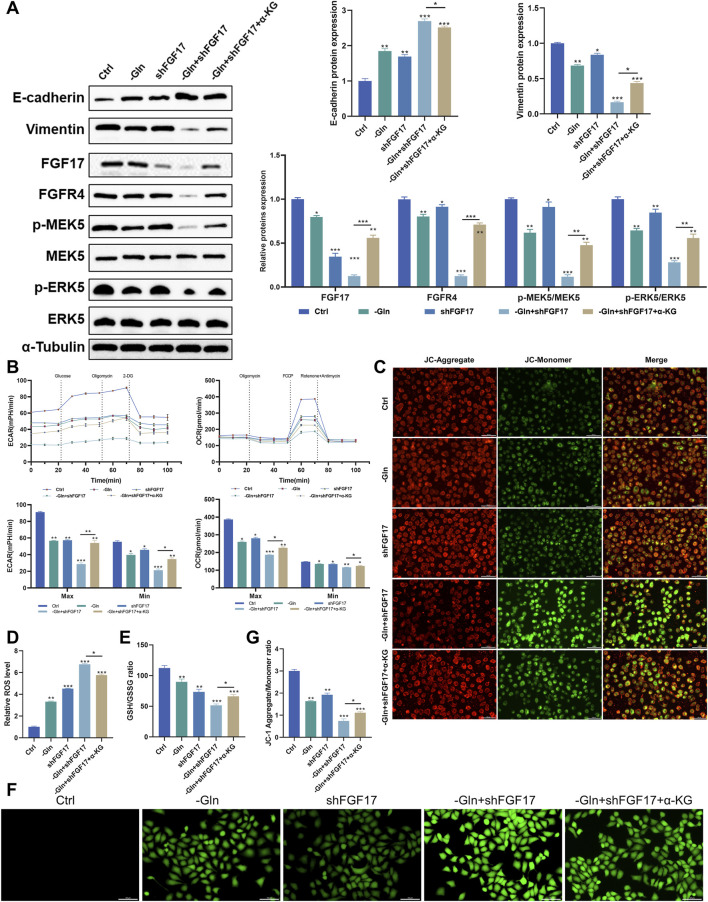
Verify that glutamine metabolism acts on MEK5/ERK5 through the FGF17-FGFR4 axis to affect the oxidative stress level and apoptosis in NCI-H1299 cells. **(A)** Western blot analysis on the protein expression of E-cadherin, Vimentin, FGF17, FGFR4, p-MEK5, MEK5, p-ERK5, and ERK5 in NCI-H1299 cells (The blot for tubulin is shown once as it serves as the loading control for both GLUL and the above proteins, which were derived from the same cell samples); **(B)** Effect of FGF17 silencing and glutamine deprivation on ECAR and OCR in NCI-H1299 cells; **(C)** JC-1 mitochondrial membrane potential assay in NCI-H1299 (scale bar = 100 μm); **(D)** Quantitative results of the JC-1 mitochondrial membrane potential assay by ImageJ; **(E)** The effects of FGF17 silencing and glutamine deprivation on GSH/GSSG levels in NCI-H1299; **(F)** DCFH-DA assay to assess ROS level in NCI-H1299 (scale bar = 100 μm); **(G)** Quantitative results of the DCFH-DA assay by ImageJ. (Quantitative data are expressed as Mean ± SD, with three samples at least per group. For comparisons with Ctrl groups, ns, no significance; *p < 0.05, **p < 0.01, ***p < 0.001).

To investigate the mechanism by which glutamine-mediated activation of the FGF17–FGFR4 axis influences invasion and migration in NCI-H1299 cells, a scientific hypothesis was proposed based on literature review and experimentally validated. As shown in Figure 4A, Western blot analysis indicated that FGF17 protein levels were markedly lower in the shFGF17 group than in the Ctrl group (p < 0.001), with a corresponding decrease in FGFR4 expression (p < 0.05). Simultaneously, phosphorylation levels of the downstream signaling molecules p-MEK5 (Ser311) and p-ERK5 (Thr218/Tyr220) were reduced (p < 0.05 or p < 0.01), indicating that FGF17 knockdown effectively blocked the oncogenic FGFR4–MEK5/ERK5 signaling axis. Moreover, the cells within the shFGF17 group exhibited a characteristic reversion of the epithelial–mesenchymal transition (EMT) phenotype. Specifically, the expression of the epithelial marker E-cadherin was notably increased (p < 0.01). In contrast, the expression of the mesenchymal marker Vimentin was decreased (p < 0.05). This indicates an improvement in intercellular adhesion and a restraint on the migratory and invasive capabilities of the cells. Metabolic rescue experiments using α-KG markedly reversed these protein expression changes. This rescue effect indicated that α-KG, as a critical metabolite of the TCA cycle, compensated for glutamine deficiency to reverse the inhibitory effect of FGF17 knockdown on the ERK5–NRF2 axis and EMT. These results confirmed that FGF17 regulates glutamine flux toward α-KG, exacerbates oxidative stress, and thereby promotes EMT progression while inhibiting tumor cell invasion and migration.

To verify the influence of this mechanism on glutamine metabolic reprogramming within NCI-H1299 cells, a Seahorse analyzer was employed to measure the extracellular acidification rate (ECAR) and oxygen consumption rate (OCR) among various intervention groups. The findings showed that shFGF17 notably enhanced both glycolysis and oxidative phosphorylation. Moreover, the addition of α-KG further boosted these two processes (Figure 4B). These findings suggested that both glutamine deprivation and FGF17 silencing caused NCI-H1299 cells to switch toward glycolytic energy production to compensate for mitochondrial dysfunction. A pronounced decline in mitochondrial membrane potential, assessed through the red/green fluorescence ratio in JC-1 staining, was observed in the shFGF17 group (p < 0.01). These results indicate pronounced mitochondrial depolarization and impaired mitochondrial function (Figures 4C,D). Furthermore, the effect of FGF17 on mitochondrial function and oxidative stress in NCI-H1299 cells was assessed. Intracellular reactive oxygen species (ROS) levels were measured using the DCFH-DA probe. ROS levels were significantly elevated in the shFGF17 group compared with the Ctrl group (p < 0.001). Concurrently, the ratio of reduced glutathione (GSH) to oxidized glutathione (GSSG) declined, suggesting a disturbance in the glutathione redox equilibrium (p < 0.001). These observations implied that the deficiency of FGF17 caused the malfunction of the cellular antioxidant system (Figures 4E–G). Collectively, these results confirmed that glutamine metabolism in NSCLC activates the FGF17/MEK5/ERK5/NRF2 signaling pathway via GLUL, thereby lowering oxidative stress levels in the tumor microenvironment and enhancing migratory and invasive capacities of tumor cells.

### shFGF17 and cisplatin suppress tumor growth and cell migration in heterotopic xenograft mouse models

Cellular functional and mechanistic studies indicated that FGF17 knockdown inhibited the FGFR4/MEK5/ERK5/NRF2 signaling axis, disrupted redox balance, and suppressed EMT progression, thereby reducing tumor cell migration and invasion. To confirm the broad applicability of this mechanism within the *in-vivo* microenvironment and in the situation of therapeutic resistance, heterotopic xenograft mouse models incorporating shFGF17 were created. This model utilized tumor cells derived from clinical patients, maintaining their original heterogeneity and cisplatin resistance, thus allowing simultaneous evaluation of the regulatory effects of FGF17 targeting on tumor growth, metabolic reprogramming, and chemosensitization.

**FIGURE 5:**
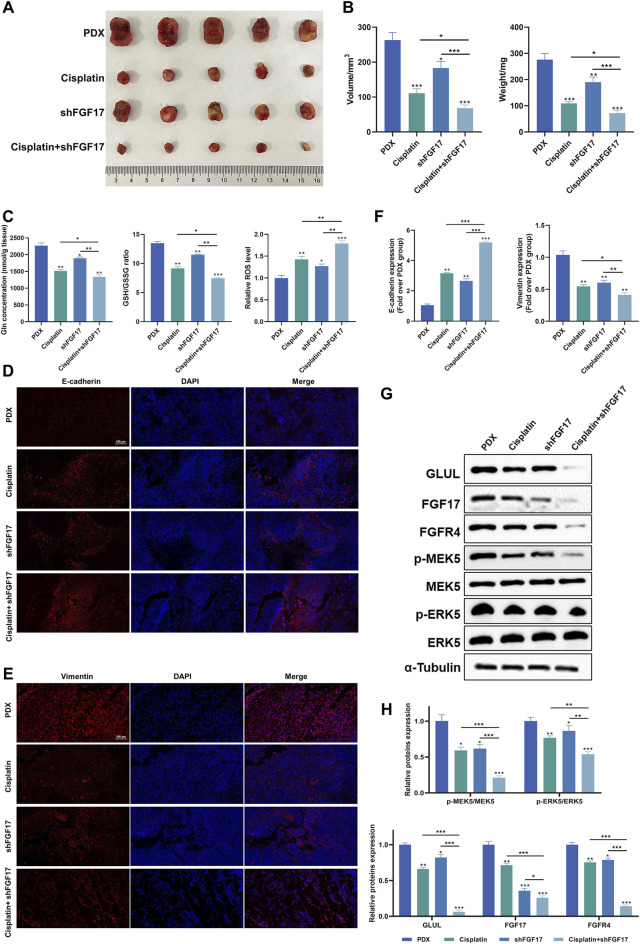
Impact of FGF17 knockdown and cisplatinum on tumor growth in tumor-bearing mice. **(A)** Dissection images of subcutaneous xenograft mice in each group (*n* = 5); **(B)** Tumor volume and weight in subcutaneous xenograft mice (*n* = 5); **(C)** The levels of glutamine, GSH/GSSG ratio, and ROS in the tumors of each group of mice; **(D)** Representative image of E-cadherin immunofluorescence (scale bar = 100 μm); **(E)** Representative image of Vimentin immunofluorescence (scale bar = 100 μm); **(F)** Quantitative results of immunofluorescence for E-cadherin and Vimentin proteins by ImageJ; **(G)** Western blot analysis on the protein expression of E-cadherin, Vimentin, FGF17, FGFR4, p-MEK5, MEK5, p-ERK5, and ERK5 in mice; **(H)** Quantitative results of Western blot analysis by ImageJ. (Quantitative data are presented as the Mean plus or minus the Standard Deviation, with a minimum of three samples for each group. When making comparisons with the PDX group, a single asterisk (*) indicates p-value less than 0.05, double asterisks (**) denote p-value less than 0.01, and triple asterisks (***) signify p-value less than 0.001).

The representative images presented in Figure 5A indicated that, when compared to the PDX group, the tumor volume decreased across all other groups. The most substantial reduction was noted in the Cisplatin + shFGF17 group. This tendency was further corroborated by the tumor volume measurements taken with calipers (Figure 5B). In addition, when contrasted with the PDX group, the tumor weights in the shFGF17 and Cisplatin groups were notably diminished (*p* < 0.001). The Cisplatin + shFGF17 group, however, showed an even more significant suppression of tumor growth (*p* < 0.001), suggesting a synergistic effect against tumors. Compared with the PDX group, both Cisplatin and shFGF17 reduced intratumoral glutamine and glutathione levels, while significantly increasing ROS accumulation, resulting in redox imbalance (Figure 5C). This finding mirrored the *in vitro* results, suggesting that in the mouse model, shFGF17 altered the oxidative stress state and glutamine metabolism of the tumor microenvironment.

Immunohistochemistry revealed that, relative to the PDX group, all treatment groups exhibited a notable elevation of E-cadherin expression (*p* < 0.01 or *p* < 0.001) and a clear reduction in Vimentin-positive regions (*p* < 0.01). Among them, changes were most pronounced in the Cisplatin + shFGF17 group, further supporting a synergistic effect and reversal of the EMT phenotype in tumor cells (Figures 5D–F). Molecular validation revealed a marked reduction of FGF17 protein levels in tumor samples from the shFGF17 group (*p* < 0.001), accompanied by concurrent suppression of its receptor FGFR4 and downstream p-MEK5 and p-ERK5 (Thr218/Tyr220) (*p* < 0.05). In addition, GLUL protein expression was reduced in both shFGF17 and Cisplatin groups (*p* < 0.05 or *p* < 0.01), and was almost completely suppressed in the Cisplatin + shFGF17 group (*p* < 0.001, Figures 5G,H). These results suggested that FGF17 may partly influence glutamine synthesis, and that cisplatin exerts its antitumor effect, at least in part, by modulating glutamine metabolism in tumor cells, with this effect amplified by FGF17 silencing.

Collectively, our findings demonstrated that in tumor cell glutamine metabolism, the GLUL-mediated FGF17/FGFR4/MEK5/ERK5 signaling axis sustains redox homeostasis in the tumor microenvironment via activation of NRF2-dependent antioxidant programs. Its silencing induces ROS bursts and synergistically enhances cisplatin-mediated inhibition of EMT progression, thereby exerting a chemosensitizing effect.

## Discussion

In this study, by employing single-cell multi-omics technologies combined with multi-level functional validation in clinical samples, cell lines, and animal models, the characteristics of glutamine metabolic reprogramming within the tumor microenvironment of NSCLC were investigated in depth. The important role and mechanistic pathways of glutamine metabolism in NSCLC were confirmed, offering insights that may facilitate the design of anti-NSCLC drugs and therapies targeting glutamine metabolism.

Single-cell RNA sequencing results from public databases demonstrated that the cellular composition of NSCLC patient tissues differed from that of normal tissues. Among the nine identified cellular subtypes, the epithelial subtype exhibited the most significant difference between normal and NSCLC tissues. Through integrated RNA sequencing analysis, fibroblast growth factor 17 (FGF17) was identified as the core target of glutamine metabolism in NSCLC tissues. Further analysis confirmed that FGF17 was highly expressed in NSCLC patients and associated with patient survival. Clinical sample analysis verified the synchronous expression of GLUL and FGF17 in patients. *In vitro* experiments with NCI-H1299 cells and *in vivo* xenograft models showed that shFGF17 elevated oxidative stress, inhibited EMT, and reduced tumor cell motility and invasiveness. The GLUL-mediated FGF17/FGFR4/MEK5/ERK5 signaling pathway was validated at both the cellular and animal levels.

The FGF family consists of 22 polypeptides and can be divided into paracrine and endocrine subfamilies (Katoh and Nakagama, 2014; Krzyscik et al., 2024). They transmit signals through four tyrosine kinase receptors (FGFR1–4) and play critical regulatory roles in embryonic development, tumor progression, tissue repair, and cellular metabolism (Xie et al., 2020; Gedaj et al., 2024; Giacomini et al., 2024). FGF17 belongs to the FGF8 paracrine subfamily, whose receptor-binding properties are highly similar. It activates high-affinity receptor FGFR4 in a paracrine manner, thereby driving malignant tumor progression (Heer et al., 2004; Katoh and Nakagama, 2014). Through bioinformatics analysis of TCGA datasets, it was discovered that FGF17 was notably over-expressed in NSCLC tissues. Patients exhibiting high FGF17 expression showed a marked reduction in overall survival (*p* < 0.05). A significant relationship was identified between FGF17 expression levels and the percentage of PD-L1-positive cells in tumor tissues, as verified by the clinical cohort study. Additionally, FGF17 levels were significantly associated with the serum tumor markers CA125 and CEA (with a correlation coefficient r ranging from 0.5 to 0.7 and *p* < 0.001). Collectively, these findings not only suggest that FGF17 is deeply involved in the dysregulated glutamine metabolism observed in NSCLC patients, but also highlight its potential as a therapeutic target. Given its significant association with key clinical biomarkers and poor prognosis, targeting FGF17 may represent a promising strategy for developing novel therapeutic interventions in NSCLC, potentially in combination with existing modalities such as immune checkpoint.

FGFR4, the key functional receptor of FGF17, is overexpressed in several cancers, including NSCLC, colorectal, breast, and hepatocellular carcinoma, and is closely linked to unfavorable patient outcomes (Lu et al., 2019; Chen et al., 2023; Braun et al., 2024). Mechanistic analyses reveal that FGFR4 signaling contributes to abnormal cell proliferation, EMT, pecific killing and drug resistance (Shen et al., 2023; Timpanaro et al., 2023; Yang et al., 2023). Studies indicate that in NSCLC, the G2041A mutation in FGFR4 can activate the MAPK, PI3K, and JAK-STAT signaling pathways, promoting tumor proliferation, survival, and immune evasion (Ramos-Ramírez et al., 2025). Ligand-bound activated FGFR4 phosphorylates and activates its substrates PLCγ and fibroblast growth factor receptor substrate 2, which in turn activate downstream signaling pathways, including MEK/MAPK, PI3K/AKT, PKC, and STATs (Wu et al., 2017; Levine et al., 2020). Among these, the MAPK pathway is commonly altered in human cancers and contributes to tumor initiation and progression by modulating key cellular processes, including proliferation, differentiation, survival, and apoptosis (Peluso et al., 2019; Drosten and Barbacid, 2020; Fang et al., 2023). As a central component of the MAPK signaling cascade, MEK can specifically activate downstream ERK, forming the functional “MEK–ERK” module to mediate signal transduction (Yu et al., 2021; Ullah et al., 2022). This mechanism has been validated in hepatocellular carcinoma models, where sustained activation of the FGFR4–MAPK pathway induced EMT, mediated immune evasion and metastasis, and enhanced tumor invasiveness (Xie et al., 2023). In recent years, the MEK5/ERK5 branch of the MAPK family has attracted attention for its unique role in tumor invasion and metastasis. Unlike the classical MEK1/2–ERK1/2 pathway, the MEK5/ERK5 pathway specifically regulates the expression of invasion-related genes such as MMP9 and VEGF through phosphorylation of transcription factors Elk-1 and MEF2C (Mehta et al., 2003; Hoang et al., 2017; Paudel et al., 2021). In breast cancer models, the MEK5–ERK5 axis was identified as a critical mediator; blocking this pathway counteracted TGFβ-induced EMT and reduced the motility and invasiveness of breast cancer cells (Pavan et al., 2018). In SCLC, the MEK5–ERK5 axis mediated lipid metabolic reprogramming through regulation of the mevalonate pathway, thereby maintaining tumor cell survival (Cristea et al., 2020). Functional experiments in the present study demonstrated that knockout of FGF17 inhibited EMT progression in NSCLC cells and significantly reduced cell migration and invasion. Taken together, a scientific hypothesis was proposed and experimentally validated: the MEK5/ERK5 pathway may constitute the key mechanistic route through which the FGF17–FGFR4 axis regulates oxidative stress levels and promotes invasion and metastasis in NSCLC.

Programmed death ligand 1 (PD-L1) interacts with PD-1 on immune cells, transmitting immunosuppressive signals and allowing tumor cells to escape immune surveillance. It has been demonstrated that PD-L1 expression levels are significantly correlated with the efficacy of immune checkpoint inhibitors (Reck et al., 2022). CA125 is a high-molecular-weight glycoprotein that has been proven to possess high specificity for lung cancer diagnosis. A study including 175 NSCLC patients and 180 individuals with benign lung lesions reported significantly elevated serum CA125 levels in NSCLC compared with benign lesions, with specificity as high as 92% (Liu, 2020; Li et al., 2021). CEA, an acidic glycoprotein of the immunoglobulin superfamily, participates in cell adhesion processes (Hammarström, 1999). As one of the most extensively studied tumor markers, CEA is widely used to assess tumor burden and metastatic potential. Studies have reported that NSCLC patients presenting lymph node metastasis had markedly elevated CEA levels compared to patients without metastasis (Liu, 2020). Nevertheless, the application of single tumor markers in NSCLC diagnosis and treatment has limitations. Combined detection of multiple markers has been shown to enhance clinical utility. For example, combined detection of CEA, CA125, and other markers such as CYFRA21-1 and NSE significantly improves diagnostic sensitivity and specificity (Liu, 2020; Xu et al., 2024). In summary, PD-L1 serves as a key indicator for guiding the use of immune checkpoint inhibitors, while CA125 and CEA, as classical serum tumor markers, are indispensable in auxiliary diagnosis, therapeutic efficacy monitoring, and prognostic assessment. Therefore, investigating the correlation of FGF17 protein with them will provide new insights into the pathological mechanisms of NSCLC. Their combined detection is expected to offer more comprehensive guidance for the clinical diagnosis, treatment monitoring, and prognostic evaluation of NSCLC, holding significant clinical implications.

Despite the use of advanced techniques and comprehensive experimental validation, this study has several limitations. First, the focus was primarily on the functions and mechanisms of glutamine-mediated FGF17 in NSCLC, but its roles in different NSCLC subtypes and across various NSCLC cell lines require further investigation. Assessing alterations in the expression of glutamine and FGF17 across various NSCLC cell lines would increase the clinical significance of these results. Second, glutamine-mediated cellular functions are complex and diverse, and its effects on tumor cell proliferation warrant further exploration. By combining single-cell metabolomic analysis and real-time assessment of metabolic fluxes, it is possible to decipher the metabolic interactions between glutamine metabolism and NSCLC cells in diverse microenvironmental settings. This process will contribute to clarifying the interplay between the FGF17-FGFR4 axis and other signaling cascades. Moreover, because of technological constraints, multiplex immunohistochemical techniques and spatial transcriptomic analysis were not utilized. This omission might have hindered a comprehensive portrayal of GLUL and FGF17 expression within the tumor microenvironment. The integration of proteomic and metabolomic approaches would enable a more comprehensive identification of the crucial molecules participating in glutamine metabolism. It would also reveal their spatial arrangement and their interactions with other cellular constituents. Consequently, this would offer more in-depth theoretical backing for understanding the pathophysiology of NSCLC. Finally, although FGF17’s role in NSCLC was confirmed using both *in vitro* and *in vivo* systems, it remains unclear whether these models accurately reflect the human NSCLC tumor microenvironment.

## Conclusion

To sum it up, this research underscored the crucial function of the FGF17-FGFR4 axis in the GLUL-driven glutamine metabolic reconfiguration of tumor cells. Moreover, it provided new insights into the metabolic patterns and aggressive behavior of cells within the NSCLC tumor microenvironment.

## Data Availability

The original contributions presented in the study are included in the article/Supplementary Material, further inquiries can be directed to the corresponding author.

## Supplementary material

SUPPLEMENTARY FIGURE S1 Impact of FGF17 knockdown and cisplatinum on tumor growth in tumor-bearing mice. **(A)** Violin graphs depicting the proportion of mitochondrial genes (percent.mito), ribosomal genes (percent.ribo), and hemoglobin genes (percent.hb) for every cell. **(B)** Scatter diagrams presenting the relationship between nCount_RNA and nFeature_RNA in the filtered dataset. **(C)** tSNE clustering dendrogram of single-cell RNA-sequencing (scRNA-seq) data. **(D)** Expression intensities of certain well-known lineage-specific marker genes across distinct clusters in normal adjacent tissue and NSCLC samples. A deeper shade of red indicates a higher average expression level, and a larger circle represents a greater number of cells expressing the gene. **(E)** Sample categorization to identify outliers. **(F)** Dispersion of the standard deviation of principal components (PCs). **(G)** Examination of the scale-free fit index (on the left) and the mean connectivity (on the right) for different soft-thresholding powers.
