# Supplementary material for: Mechanistic study of glutamine metabolic reprogramming driving non-small cell lung cancer progression via the FGF17-FGFR4 axis mediating epithelial-mesenchymal transition
Source: Front Mol Biosci. 2026 Jan 2;12:1728698. doi: 10.3389/fmolb.2025.1728698 (PMC12807982; doi:10.3389/fmolb.2025.1728698)
Supplement: Supplementary file 1 [file Table1.docx]

**Supplementary Table 1 Top 8 in network ranked by MCC method.**

| Rank | Name | Score |
| --- | --- | --- |
| 1 | FGF17 | 3.0 |
| 1 | AXIN2 | 3.0 |
| 1 | HOXC5 | 3.0 |
| 1 | RHBDL3 | 3.0 |
| 1 | GAS2 | 3.0 |
| 6 | FOXD4L1 | 3.0 |
| 6 | EFCAB6 | 3.0 |
| 6 | LRRC4C | 3.0 |
