# Supplementary figures and images for: Mechanistic study of glutamine metabolic reprogramming driving non-small cell lung cancer progression via the FGF17-FGFR4 axis mediating epithelial-mesenchymal transition
Source: Front Mol Biosci. 2026 Jan 2;12:1728698. doi: 10.3389/fmolb.2025.1728698 (PMC12807982; doi:10.3389/fmolb.2025.1728698)

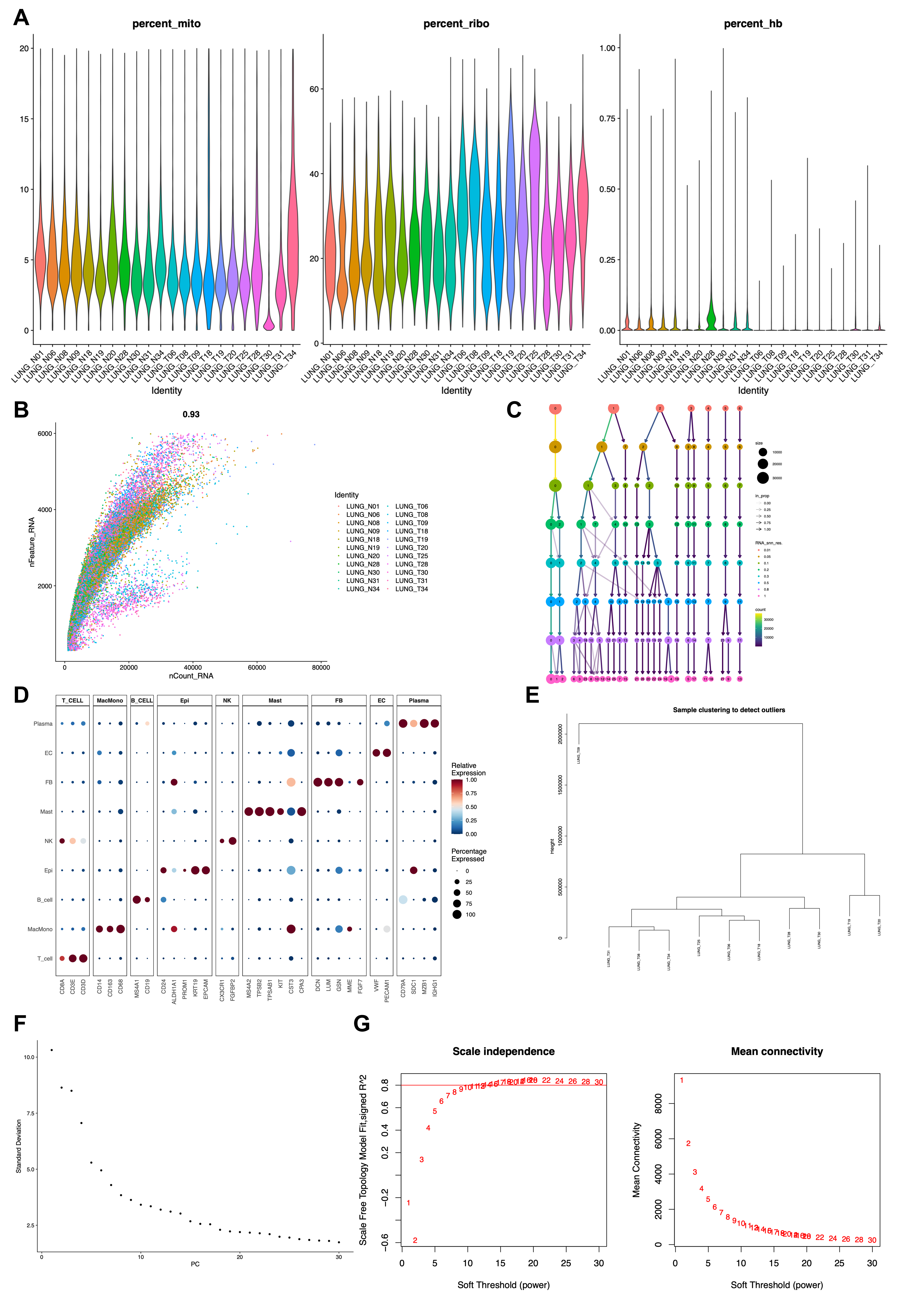

Supplement: Supplementary file 2 [file Image1.tif]
